# COVID-19 and myocardial injury: Targeting elevated biomarkers for potential novel therapies

**DOI:** 10.1016/j.clinsp.2024.100473

**Published:** 2024-08-28

**Authors:** Pengyang Li, Qun Chen, Ion S. Jovin, Anit Mankad, Jose F. Huizar, John D. Markley, Bradley Bart, Brack Hattler, Edward Lesnefsky, Edward O. McFalls

**Affiliations:** a Virginia Commonwealth University, Richmond VA; b McGuire VA Medical Center, Richmond VA; c Minneapolis VA Medical Center, Minneapolis MN; d Denver VA Medical Center, Denver CO

**Keywords:** Troponin, Myocardial injury, COVID, Outcomes, BNP, C-reactive protein

## Abstract

**Background:**

The prevalence of COVID-19 as the primary diagnosis among hospitalized patients with myocardial injury has increased during the pandemic and targeting elevated oxidant stress and inflammatory biomarkers may offer a potential role for novel therapies to improve outcomes.

**Methods:**

At a single VA Medical Center from January 1 through December 31, 2021, troponin assays from patients being evaluated in the Emergency Room for consideration of admission were analyzed and peak levels from each patient were considered abnormal if exceeding the Upper Reference Limit (URL). Among admitted patients with an elevated troponin level, ICD-10 diagnoses were categorized, biomarker elevations were recorded, and independent predictors of death in patients with COVID-19 were determined at a median of 6-months following admission.

**Results:**

Of 998 patients, 399 (40 %) had a negative troponin and were not included in the analysis. Additional patients with an elevated troponin were also excluded, either because they were not admitted (*n* = 68) or had a final diagnosis of Type 1 MI (*n* = 117). Of the remaining 414 patients with an elevated peak troponin, COVID-19 was the primary diagnosis in 43 patients (10 %) and was the 4^th^ most common diagnosis of patients admitted with myocardial injury behind congestive heart failure, sepsis, and COPD or pneumonia. At a median of 6-months following admission, 18 (42 %) of the COVID-19 patients had died and independent predictors of death (*Odd Ratio*: Confidence Intervals) were age (1.18: 1.06‒1.37), Troponin level (Log 10 transformed) (16.54: 2.30‒266.65) and C-Reactive Protein (CRP) (1.30: 1.10‒1.65).

**Conclusions:**

Newly diagnosed COVID-19 during the pandemic was a common cause of elevated troponin in hospitalized patients without a Type 1 MI. Age, peak troponin level and peak CRP level were independent predictors of poor outcomes and suggest a need to target these cardiac biomarkers, potentially with novel antioxidant or anti-inflammatory therapies.

## Highlights

• During the COVID-19 pandemic, the prevalence of COVID-19 as the primary diagnosis for hospitalized patients with myocardial injury was 10 %.
• •The mortality at 6 months was more than 40 % and independent predictors of death included age, peak troponin, and peak C-reactive protein levels.
• Future studies should focus on targeting elevated oxidant stress and inflammatory biomarkers among these patients.

## Introduction

During the COVID-19 pandemic, the number of patients being evaluated for consideration of Acute Coronary Syndrome (ACS) has increased. The utility of troponin assays, as a cardiac biomarker for optimal risk-stratification of patients with unstable cardiac symptoms is important for timely revascularization in suitable patients.^1^, ^2^, ^3^ Although troponin assays have value for the diagnosis of a Myocardial Infarction (MI), their application has extended to a heterogeneous cohort of patients,^4^^,^^5^ creating a confusing conundrum for providers.^6^ Based on a consensus statement, the diagnosis of MI in patients with an elevated cardiac troponin requires one or more clinical correlates of ischemia.^7^ If a primary event, it is categorized as a Type 1 MI, and if secondary to a supply-demand mismatch, is categorized as a Type 2 MI. In the absence of ischemia, the event is designated myocardial injury. Among patients with myocardial injury and a Type 2 MI, adverse outcomes are high, compared with those patients with a Type 1 MI.^8^, ^9^, ^10^, ^11^, ^12^, ^13^, ^14^

COVID-19 has become a common cause of myocardial injury among hospitalized patients who are not candidates for coronary interventions. Elevated cardiac biomarkers are an important identifier of adverse outcomes^15^, ^16^, ^17^, ^18^, ^19^, ^20^ and may offer an opportunity to advance novel therapies. To assess the impact of COVID-19 on patients with myocardial injury, the authors did an analysis of all patients getting a troponin assay at the Richmond VA Medical Center in 2021 and focused on those hospitalized patients with an elevated troponin level who did not have an ACS or Type 1 MI. The authors hypothesized that during this COVID-19 pandemic, the prevalence of COVID-19 as the primary diagnosis comprised a high percentage of all patients being admitted for observation, and biomarkers predicted poor outcomes following admission.

## Methods

The study focused on all consecutive patients presenting to the Emergency room at the Richmond VA Medical Center for any reason and subsequently had a troponin assay drawn between January 1 and December 31, 2021. The primary focus was hospitalized patients with a non-ACS condition. Accordingly, those patients who were either not admitted to the hospital or were diagnosed with ACS or a Type 1 MI were excluded from the analysis. The study was approved by the IRB at the Richmond VA Medical Center (IRB #1575619) and the expression of the data followed the STROBE Statement. The present analysis of all troponins obtained utilized a Siemens 4^th^ generation troponin assay and the peak troponin level was identified in all patients as defined by the Upper Reference Limit (URL) of the assay (0.045 ng/mL). In addition to troponins, peak levels of biomarkers that were also obtained included NT-proBNP and C-Reactive Protein. ICD-10 diagnoses were obtained from the discharge summary and categorized according to the primary diagnosis.

### Statistical analysis

Survival was determined at a median of 6 months following hospital discharge. Continuous variables were expressed as a mean with Standard Deviation (SD) and were tested with a *t*-test, while categorical variables were expressed as percentages and tested with a Chi-Square test. To assess the prognostic performance of biomarkers, a multivariate logistic regression model introducing peaked troponin and C-Reactive protein as continuous variables adjusted for age was conducted.

## Results

**Fig. 1:**
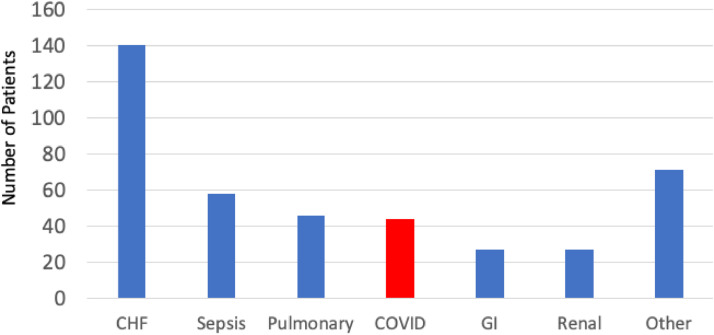
An analysis of Veterans admitted through the Emergency Room at the Richmond VA Medical Center in 2021 shows that 10 % of the individuals with myocardial injury and a non-ACS diagnosis had COVID-19 as the primary diagnosis, with a poor outcome at a median of 6-months following hospital discharge. CHF, Other diagnoses included Congestive Heart Failure; COPD or Pneumonia, Pulmonary; GI, Gastrointestinal from bleeding, renal failure or other non-specific diagnoses.

The analysis included a total of 2,640 troponin assays which were obtained in 998 patients evaluated in the Emergency Room. Among patients with multiple assays, the peak troponin value was used for this analysis. Negative test results were defined as those results that did not exceed the Upper Reference Limit (URL) of the assay and were present in 399 (40 %) patients. They were censored from the analysis. An additional 68 patients with elevated troponin were not admitted for observation and were also censored from analysis. Of the remaining 531 patients who were admitted with a peak troponin level that exceeded the URL of the troponin assay, 117 (22 %) had a primary diagnosis of Type 1 MI, and they were also censored from additional analyses. The remaining 414 patients with an elevated peak troponin had another primary diagnosis for admission, with a secondary diagnosis by exclusion, of either a Type 2 MI or myocardial Injury. The primary admitting diagnosis of all patients admitted with an elevated troponin and a non-Type 1 MI was COVID-19 in 43 of the 414 patients (10 %) and was the 4^th^ most common diagnosis behind congestive heart failure (*n* = 140), sepsis (*n* = 58) and a pulmonary problem from either COPD or pneumonia (*n* = 46) (Fig. 1).

**Fig. 2:**
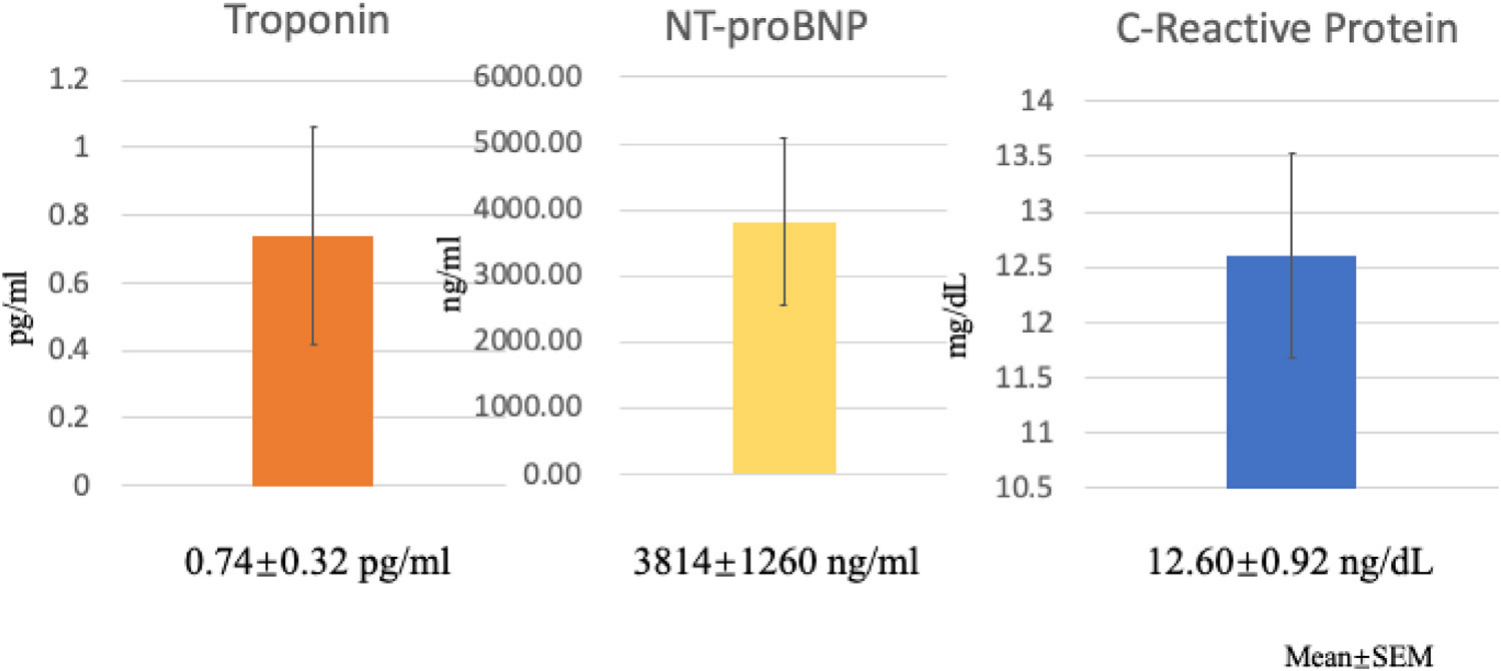
Peak levels of troponin, NT-proBNP and C-reactive Protein are shown for all patients admitted with newly diagnosed COVID-19 and elevated troponin levels (Data are expressed as Means and SEM).

In addition to troponin levels, which were elevated in all patients with COVID-19, additional clinical biomarkers that were associated with poor outcomes included NT-proBNP and C-Reactive Protein levels (Fig. 2). Of note, although troponin and CRP levels were available in all 43 COVID-19 patients during their hospitalization, NT-proBNP levels were not available in 10 of the 43 COVID-19 patients. At a median of 6 months following hospital admission, 18 of the 43 (42 %) patients with COVID-19 had died. These data underscore the high prevalence of newly diagnosed COVID-19 patients admitted with myocardial injury during the pandemic and highlight their poor long-term outcomes. All baseline characteristics that discriminated between survivors and non-survivors are shown in Table 1 and demonstrate the importance of age as an important identifier of risk for non-survival. By a logistic regression analysis, independent predictors of death were age, peak troponin levels and peak C-reactive protein levels (Table 2). These data underscore the importance of initial cardiac and inflammatory biomarkers, for identifying those individuals at risk following hospital discharge.

**Table 1:** Baseline Characteristics of COVID-19 patients admitted with myocardial injury.

	**Non-Survivors** **(***n* = **19)**	**Survivors** **(***n* = **24)**	**p-value**
**Clinical Variables**			
Age	76.5 ± 9.5	70.1 ± 8.9	0.031
Female	0 (0 %)	3 (13 %)	0.242
White	9 (47 %)	10 (42 %)	0.775
CAD	8 (42 %)	10 (42 %)	1.0
PCI/CABG	7 (37 %)	8 (33 %)	1.0
CHF	5 (26 %)	7 (29 %)	1.0
LVEF	55 ± 9	47 ± 14	0.076
Atrial fibrillation	3 (16 %)	1 (4 %)	0.328
CVA/TIA	3 (16 %)	3 (13 %)	1.0
Dementia	4 (21 %)	0 (0 %)	0.040
DM	12 (63 %)	17 (71 %)	0.748
Cancer	6 (32 %)	4 (17 %)	0.289
**Laboratory Values**			
Troponin	1.44 ± 3.00	0.18 ± 0.21	0.084
NT Pro BNP	6140 ± 10091	1876 ± 2462	0.131
LDL	58.9 ± 26.8	78.7 ± 34.4	0.052
HDL	43.9 ± 10.4	42.1 ± 10.9	0.604
Triglyceride	140.9 ± 99.9	140.5 ± 99.7	0.991
Hemoglobin A1C	7.1 ± 1.7	7.3 ± 1.9	0.696
Creatine	2.1 ± 1.5	2.0 ± 1.9	0.769
GFR	63.7 ± 82.9	56.9 ± 34.5	0.751
Albumin	2.8 ± 0.7	2.8 ± 0.5	0.783
Sedimentation Rate	73.7 ± 26.6	59.2 ± 34.1	0.181
Hemoglobin	12.4 ± 2.6	12.7 ± 2.4	0.705
C-reactive protein	15.5 ± 5.2	10.3 ± 5.4	0.004

Death at a median of 6-months following admission; Means ± Standard Deviation. CAD, Coronary Artery Disease; CABG, Prior coronary artery revascularization with PCI or Coronary Artery Bypass Surgery; LVEF, LV Ejection Fraction; DM, Diabetes Mellitus; GFR, Glomerular Filtration Rate.

**Table 2:** Odds ratio for mortality during follow-up.

**Variables**	**OR**	**95** % **CI**	**p-value**
Trop^a^	16.54	2.30, 266.65	0.020
AGE	1.18	1.06, 1.37	0.011
CRP	1.30	1.10, 1.65	0.010

a Log 10 transformed. By logistic regression model, independent predictors of death at a median of 6-months following admission are shown, along with hazard ratio and 95 % Confidence Intervals.

## Discussion

**Fig. 3:**
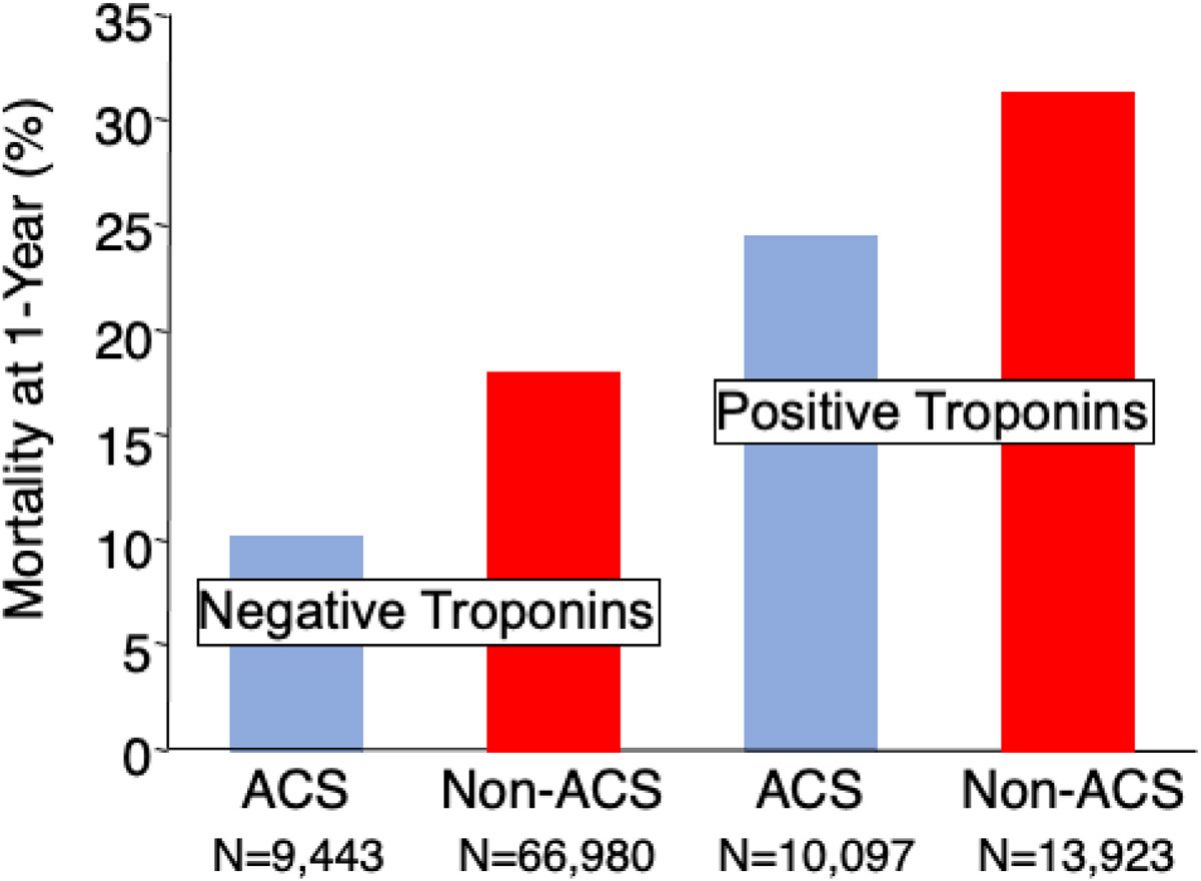
In an analysis of Veterans, a poor long-term outcome was noted among those patients with an elevated troponin level compared with those patients without an elevated troponin.^22^ Among those patients with a primary diagnosis other than Acute Coronary Syndrome, long-term mortality exceeded that of patients with the diagnosis of ACS. Data are reformatted and reproduced with permission.

The principal finding of this analysis is that newly diagnosed COVID-19 infections during the COVID pandemic accounted for more than 1 in 10 patients admitted through the Emergency Room with an elevated troponin level and a non-ACS diagnosis. The troponin molecule is specific to the heart and governs the interaction between actin and myosin cross-bridging during contraction and as such, is an accurate assay for detecting myocyte damage. Despite the value of troponin assays in the risk-stratification of patients with an ACS, their application has extended to a heterogeneous cohort of patients who will not require urgent revascularization.^4^^,^^5^ Although the authors did not discriminate between myocardial injury and a Type 2 MI in the present analysis, these diagnostic categories are often arbitrary and misclassified on post-hoc analyses, with equally poor outcomes following discharge.^8^, ^9^, ^10^, ^11^, ^12^, ^13^, ^14^ Consistent with those observations, the authors found that 42 % of the patients with COVID-19 had died at 6-months and were predicted by their peak cardiac biomarker levels, including troponin, and CRP. Among the entire cohort in this study, the prevalence of a Type 1 MI was 22 % of all patients being admitted for consideration of an ACS, underscoring the observations that the prevalence of non-Type 1 conditions with myocardial injury is increasing with the aging population.^21^ In an analysis of over 25,000 Veterans admitted to VA Medical Centers with elevated troponin levels in 2006, 43 % had a diagnosis of ACS or Type 1 MI (Fig. 3),^22^ demonstrating that myocardial injury has become more prevalent in the past 1‒2 decades, particularly during the COVID pandemic. In that study, outcomes were substantially different between patients with and without an ACS with wide variability of the primary diagnoses defined at discharge. In the recent era with ICD-10 diagnostic codes, the prevalence of a Type 1 MI is much lower than either a Type 2 MI or Myocardial Injury, with far worse outcomes in the latter groups both early and late following hospital discharge.^12^^,^^21^^,^^23^ Considering the increased prevalence of these events, novel approaches are needed to improve outcomes.

One unifying concept that has not yet been advanced to these patients with myocardial injury and a non-ACS presentation is the potential therapeutic approaches to mitigate oxidant stress and inflammatory signals that might be responsible for future adverse events. NT Pro BNP is a modifiable biomarker in patients with congestive heart failure and has been shown to be an important marker of illness for these subsets of patients during the COVID-19 pandemic, even in the absence of heart failure.^16^, ^17^, ^18^^,^^24^ Elevated troponins are also common in these patients and predict poor outcomes.^20^ Among 416 patients hospitalized for COVID-19, 82 (20 %) had an elevated cardiac troponin level which was an independent predictor of respiratory failure and death.^25^ Although the mechanism of COVID-19-related deaths is unclear, an overarching hypothesis involves a cytokine storm, induced by enhanced IL-6 signaling.^26^^,^^27^ In an analysis of patients admitted to an acute care facility during the Wuhan epidemic, those patients admitted to the ICU had higher levels of TNF-alpha compared with non-ICU patients and a greater degree of IL-6 expression compared with the general population.^15^ Targeting IL-6 with monoclonal antibodies to prevent troponin and NT pro-BNP release has shown favorable results.^28^

Although NT-proBNP levels were higher in non-survivors compared with survivors, the differences did not reach statistical significance. Of note, 10 of the 43 patients did not get BNP assays during their hospitalization which may explain the variance with other studies. NT-proBNP has been shown to be an important cardiac biomarker in larger studies and is elevated in patients with COVID-19 induced myocardial injury, suggesting a common mechanism of release within the heart.^16^^,^^17^^,^^29^, ^30^, ^31^ In support of this, BNP and Troponin levels are increased in patients presenting with sepsis and the elevated cardiac biomarkers correlate well with increased C-reactive protein and TNF-alpha levels.^32^ The authors have shown that NT-proBNP predicts adverse outcomes in high-risk patients undergoing vascular surgery and can be reduced with preoperative administration of ubiquinone.^33^ Generalizing these findings to COVID-19 may prove rational, considering that BNP predicts adverse outcomes with COVID-19.^31^

It is conceivable that the administration of ubiquinone to patients with COVID-19 will provide important antioxidant protection within the tissue that reduces the cardiac and inflammatory biomarkers. In support of this approach, serum levels of CoQ_10_ are decreased in hospitalized patients with chronic inflammatory conditions and the reduced levels predicted risk of adverse outcomes.^34^ Lower levels are also inversely correlated with elevated inflammatory biomarkers in patients with acute diseases such as influenza.^35^ This observation is important for interpreting the results of the Q-SYMBIO trial, which among patients with stable heart failure, tested the long-term benefit of chronic administration of CoQ_10_ (300 mg/day) versus placebo. The trial was a double-blind, randomized controlled trial and demonstrated that treatment reduced long-term major cardiovascular endpoints and improved short-term functional status.^36^^,^^37^ A meta-analysis of patients with congestive heart failure who were randomly assigned to treatment with CoQ_10_ also showed improvement in functional status.^38^ In the present study, peak C-reactive protein levels on admission were an important identifier of poor outcomes, consistent with the findings of previous studies.^19^ Among patients presenting with acute coronary syndrome and undergoing coronary interventions, pre-procedural C-reactive protein is an independent predictor of readmission to the hospital within 6 months of hospital discharge.^39^^,^^40^ Clearly, targeting inflammation as a way of improving outcomes in patients with cardiovascular diseases is an important initiative.^41^ In fact, among patients with a recent myocardial infarction and an elevated high sensitivity C-reactive protein (≥ 2.0 mg%), the Canakinumab Anti-Inflammatory Thrombosis Outcomes Study (CANTOS) trial demonstrated that inhibition of Interleukin-1β (IL-1β) reduces the composite of adverse vascular events and mortality.^42^ Canakinumab is a monoclonal antibody that inhibits the release of C-reactive protein, by blocking IL-1β and the subsequent release of IL-6.^43^ Among consecutive hospitalized patients, those with an elevated C-reactive protein identify an increased risk of readmission to the hospital, when normalized to albumin ratios and blood glucose levels.^44^ This underscores the important relationship between nutritional status and metabolic syndrome, which may complicate the early postoperative recovery period with risks of subsequent infections. In support of our observations, an elevated CRP level prior to cardiac surgery is an important predictor of adverse outcomes following hospital discharge^45^, ^46^, ^47^ and among patients undergoing vascular surgery, predicts early graft failure.^48^ Among large groups of patients with known vascular disease, there is a growing awareness that an elevated CRP level is an important identifier of recurrent vascular events, and potentially modifiable with newer, novel anti-inflammatory regimens.^49^, ^50^, ^51^, ^52^

## Conclusions

In summary, myocardial injury has become more prevalent during the COVID-19 pandemic, and with the aging population presenting to urgent care facilities coupled to the advent of high-sensitivity troponin assays, will only increase in the future. Targeting cardiac biomarkers such as C-reactive Protein and NT-proBNP with specific agents that reduce oxidant stress and inflammatory signals may reduce the economic burden associated with high rates of readmission and poor quality of life measures.

## CRediT authorship contribution statement

**Pengyang Li:** Conceptualization, Formal analysis. **Qun Chen:** Writing – review & editing. **Ion S. Jovin:** Writing – original draft. **Anit Mankad:** Conceptualization. **Jose F. Huizar:** Writing – original draft. **John D. Markley:** Writing – original draft. **Bradley Bart:** Writing – original draft. **Brack Hattler:** Writing – original draft. **Edward Lesnefsky:** Writing – original draft. **Edward O. McFalls:** Conceptualization, Formal analysis, Writing – original draft.

## Declaration of competing interest

The authors declare no conflicts of interest.
